# Synthesis of Heteroaromatic Compounds by Oxidative Aromatization Using an Activated Carbon/Molecular Oxygen System

**DOI:** 10.3390/molecules14083073

**Published:** 2009-08-14

**Authors:** Yuka Kawashita, Masahiko Hayashi

**Affiliations:** Department of Chemistry, Graduate School of Science, Kobe University, Kobe 657-8501, Japan; E-mail: mhayashi@kobe-u.ac.jp (Y.K.)

**Keywords:** oxidation, aromatization, heteroaromatic compound, activated carbon

## Abstract

A variety of heteroaromatic compounds, such as substituted pyridines, pyrazoles, indoles, 2-substituted imidazoles, 2-substituted imidazoles, 2-arylbenzazoles and pyrimidin-2(*1H*)-ones are synthesized by oxidative aromatization using the activated carbon and molecular oxygen system. Mechanistic study focused on the role of activated carbon in the synthesis of 2-arylbenzazoles is also discussed. In the final section, we will disclose the efficient synthesis of substituted 9,10-anthracenes *via* oxidative aromatization.

## 1. Introduction

### 1.1. Activated carbon—O_2_ oxidation system; How we discovered it!

**Scheme 1:**
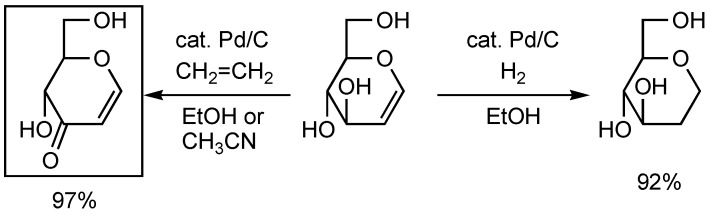
Hydrogen transfer reaction using Pd/C–ethylene system.

The oxidation of alcohols to carbonyl compounds is one of the most fundamental reactions in organic synthesis [1]. In 1999, we first reported a Pd/C—ethylene system for the oxidation of benzylic and allylic alcohols to the corresponding ketones [2,3,4,5]. A representative example of this reaction is shown in Scheme 1. Treatment of D-glucal with a catalytic amount of Pd/C in ethanol under a hydrogen atmosphere gave “normal” hydrogenated 2-deoxy-1,5-anhydroglucitol in 92% yield. In contrast, when the same reaction was carried out under an ethylene atmosphere, dehydrogenated 1,5-anhydrohex-1-en-3-ulose was obtained in 97% yield [6].

**Scheme 2:**
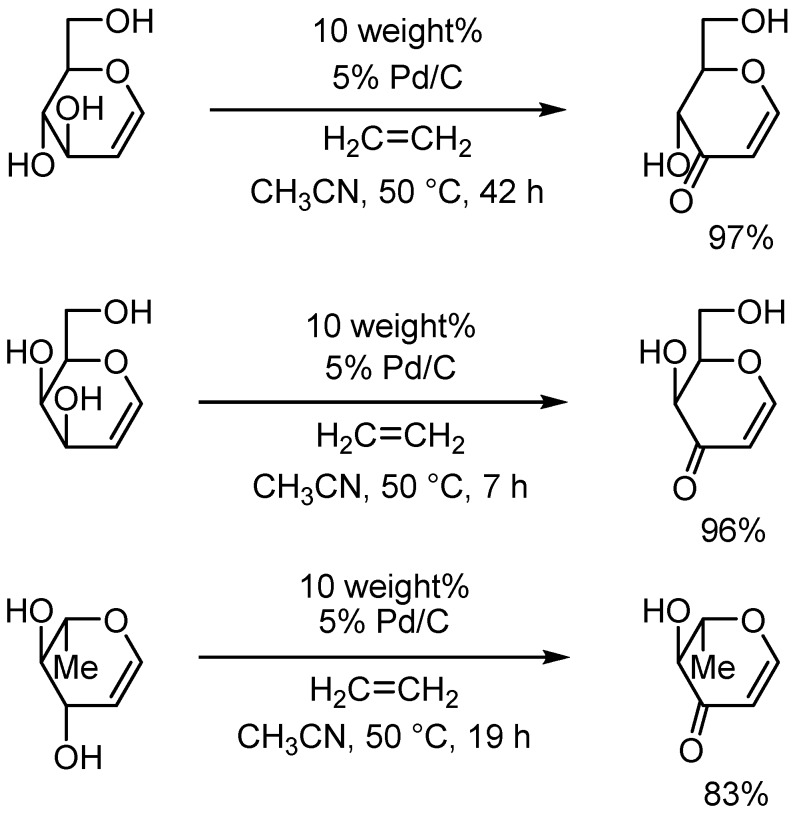
Oxidation of some glycols to enones.

The Pd/C-ethylene system also proved to be efficient for oxidation for some glycals such as D-glucal, D-galactal and L-rhamnal, as shown in Scheme 2.

This dehydrogenation, i.e., oxidation, was general for benzylic and allylic alcohols. A variety of benzylic and allylic alcohols were converted into the corresponding ketones with the aid of 20 weight% (wt%) or 50 wt% of 10% Pd/C under an ethylene atmosphere [7]. Because there is no oxygen source in this system, theoretically oxide compounds, such as nitrogen oxides and sulfoxides cannot be produced. This is the chief advantage of this oxidation system.

**Scheme 3:**
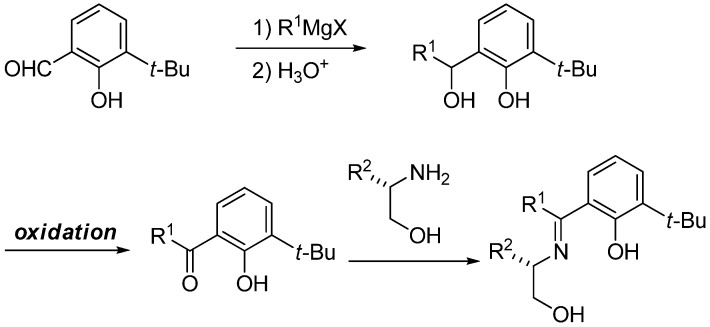
Synthesis of chiral Schiff bases.

**Scheme 4:**
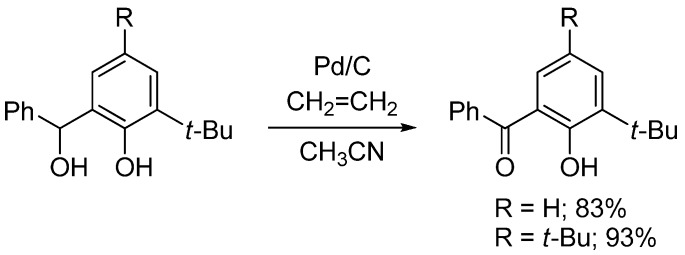
Preparation of substituted salicylketones.

During the course of our study of chiral Schiff base ligands in catalytic asymmetric reactions [8,9,10,11,12,13,14,15,16], we noted that in order to synthesize novel chiral Schiff base ligands derived from ketoimine-type substrates, there was a need to develop a new and efficient method of oxidation for benzylic alcohols after the introduction of an R^1^ group by the Grignard reaction (Scheme 3) because treatment of phenolic compounds with the conventional chromium oxide (CrO_3_) reagent produced undesired quinolic compounds and their polymerized compounds, that led to only low yields (20–30%) of the desired ketones. Therefore, at first we examined the use of our Pd/C–ethylene system for this selective oxidation of allylic alcohols in the presence of phenolic alcohols and obtained successful results (Scheme 4) [17]. We carefully studied this reaction and fortunately found that the Pd/C system can be replaced by a C (activated carbon) and oxygen system. It was surprising that Pd is not necessary but only activated carbon was required (Table 1) [18].

**Table 1:** Oxidation of benzylic alcohols using the activated carbon-O_2_ system [18]. ^a^

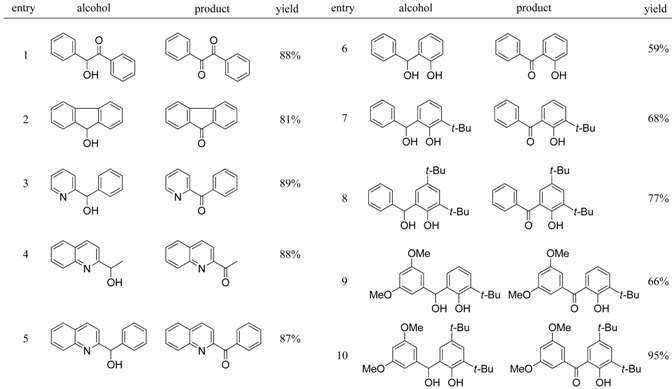

^a^ all reactions were carried out in xylene (10 mL) at 120 °C for 12 h using 500 mg of alcohol and 50 wt% of activated carbon (Shirasagi KL).

It should be noted again the same oxidations in Table 1 can also be carried out using the Pd/C—etylene system [2,3,4,5].

## 2. Results and Discussion

### 2.1. Oxidative aromatization of Hantzsch 1,4-dihydropyridines and 1,3,5-trisubstituted pyrazolines promoted by the Activated-Carbon—O_2_ system *[19,20]*

At the same time, we focussed our attention on the oxidative aromatization of dihydroaromatic compounds using the activated carbon—O_2_ system. Six- and five-membered heterocycles, such as pyridine and pyrazole moieties, are important constituents that are often found in various bioactive compounds. We first examined the efficient oxidative conversion of Hantzsch 1,4-dihydropyridines, which can be easily synthesized by the reaction of aldehydes, β-keto esters, and ammonia, into the corresponding pyridine derivatives, as shown in Table 2.

**Table 2:** Oxidative aromatization of Hantzsch 1,4-dihydropyridines with molecular oxygen promoted by activated carbon. ^a^

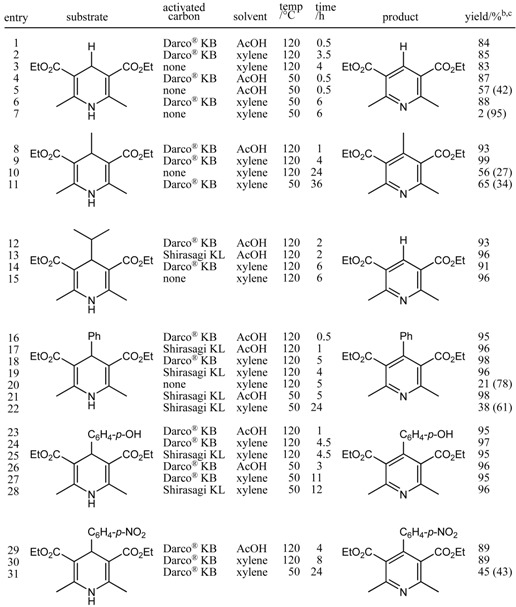

^a^ Reactions were carried out using 50 wt% of activated carbon under oxygen atmosphere; ^b^ Isolated yield after silica-gel column chromatography; ^c^ ^1^H-NMR analysis; Values in parentheses indicate the yields of recovered starting.

We discovered that to promote the oxidative aromatization of Hantzsch 1,4-dihydropyridines to pyridines, the presence of activated carbon is crucial, whereas palladium is not necessary, as the reaction proceeded with only the former and no palladium source. When the dihydropyridines were treated with 50 wt% activated carbon under an oxygen atmosphere in either acetic acid or xylene, the desired pyridines were produced in excellent yields. For example, treatment of diethyl 1,4-dihydro-2,6-dimethyl-3,5-pyridinedicarboxylate with 50 wt% activated carbon (Darco^®^ KB) under an oxygen atmosphere at 120 ºC in acetic acid for 30 min afforded the corresponding pyridine in 84% yield (Table 2, entry 1). 

**Table 3:** Oxidative aromatization of 1,3,5-trisubstituted pyrazolines with molecular oxygen promoted by activated carbon.^a^

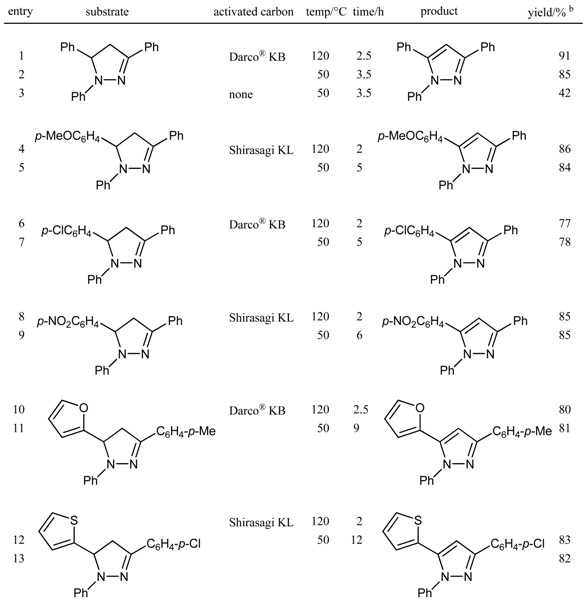

^a^ All reactions were carried out using 50 wt% of activated carbon in acetic acid under an oxygen atmosphere; ^b^ Isolated yield by column chromatography.

The reaction was found to occur even at 50 ºC (entry 4). This transformation also proceeded smoothly in xylene (120 ºC, 3.5 h, 85%; 50 ºC, 6 h, 88%; entries 2 and 6). Less time was required when acetic acid was used as the solvent compared to xylene, but the yields were similar. Two types of activated carbon were used in these transformations. One was Darco^®^ KB, and the other was Shirasagi KL. Their reactivity proved to be almost the same. The reaction also proceeded in some cases, albeit in lower yields, even without activated carbon (entries 3 and 15). Table 2 lists the conversion of Hantzsch 1,4-dihydropyridines possessing a variety of substituents, such as H, Me, *i*-Pr, Ph, *p*-OH-C_6_H_4_, and *p*-NO_2_C_6_H_4_, at the 4-position to the corresponding pyridine derivatives. The reaction of a Hantzsch 1,4-dihydropyridine bearing an isopropyl group at the 4-position provided a dealkylated pyridine (entries 12–15). This phenomenon is consistent with that described in previous reports [21,22].

Next, we applied the activated carbon—O_2_ system to the aromatization of 1,3,5-trisubstituted pyrazolines to the corresponding pyrazoles (Table 3). The system also exhibited high performance in this transformation. 1,3,5-Trisubstituted pyrazolines bearing a variety of substituents were treated with 50 wt% activated carbon (Darco^®^ KB or Shirasagi KL) at 120 ºC or 50 ºC in acetic acid for 2–12 h to afford the corresponding pyrazoles in high yields (77–91%). From entries 2 and 3 in Table 3, it is clear that activated carbon promotes the conversion of pyrazolines to pyrazoles. 1,3,5-Trisubstituted pyrazolines are readily prepared from chalcone derivatives and phenylhydrazine. Therefore, the aromatization of pyrazolines provides easy access to pyrazole derivatives that also exhibit biological activities, including antipyretic, anticonvulsant, and analgesic properties. 

Thus, we have developed an extremely practical and facile method for the aromatization of Hantzsch 1,4-dihydropyridines and 1,3,5-trisubstituted pyrazolines with molecular oxygen promoted by activated carbon. This method is not only environmentally friendly but also economical and operationally simple: only oxygen and readily available and inexpensive activated carbon are used, and neither metal oxides nor organic oxidizing agents are necessary. 

### 2.2. Synthesis of substituted indoles by oxidative aromatization using the activated carbon–—molecular oxygen system *[23]*

The indole moiety occurs naturally in a variety of structures. Many synthetic methods are available to obtain the indole framework. The Fischer indole synthesis is one of the most classical and established methods. Other methods include the Reissert synthesis, Gassman synthesis, Bartoli synthesis, Madelung synthesis, Nenitzescu synthesis, Larock synthesis, and their modified versions. All of these include a cyclization step. An alternative method is the dehydrogenation of indolines to indoles [24,25,26]. Recently, Kaneda and coworkers reported the dehydrogenation of indolines to indoles using a hydroxyapatite-bound palladium catalyst (toluene, under argon, 100 ºC) [27]. Here, we report an efficient synthesis of substituted indoles and benzazoles by oxidative aromatization using the activated carbon—molecular oxygen system. Benzazoles unsubstituted at the 2-position are also versatile compounds because they have the potential for the introduction of some substituents at the 2-position. For example, Bellina and Rossi have reported the palladium- and copper-mediated C-2 arylation of benzazoles [28].

First, we examined the effect of the amount of activated carbon (Shirasagi KL, Japan EnviroChemicals, Ltd.) in xylene at 80 ºC for 9 h. When activated carbon was absent, only 6% of indole was obtained from indoline. The yield was increased to 89% in the presence of 100 wt% activated carbon. When the reaction was carried out under an argon atmosphere, indole was obtained in 16% yield. This may be attributed to the existence of oxygen in the activated carbon. The reaction proceeded even under an air atmosphere (78%). When the reaction was performed in the presence of molecular oxygen, 89% of indole was obtained. Using optimized conditions, a variety of indoline derivatives were oxidized to the corresponding indoles. The results are summarized in Table 4. In all cases examined, Shirasagi KL was more effective than Darco^®^ KB.

**Table 4:** Oxidative conversion of substituted indolines into indoles. ^a^ 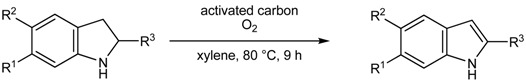

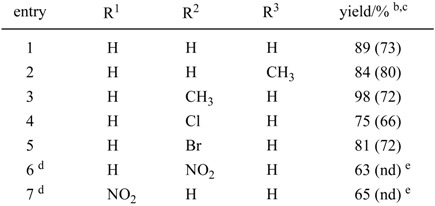

^a^ All reactions were carried out using 100 weight% of Shirasagi KL in xylene at 80 °C for 9 h; ^b^ Isolated yield after silica-gel column chromatography; ^c^ The values in the parentheses are yields when Darco KB was used instead of Shirasagi KL as the activated carbon; ^d^ Isolated yield after rescrystallization; ^e^ nd= no data.

### 2.3. Synthesis of 2-arylimidazoles by oxidation of 2-arylimidazolines using the activated carbon—molecular oxygen system *[29]*

Substituted imidazoles are important moieties constituting pharmaceuticals, pesticides, and bioactive compounds. Recently, imidazoles have found a use as skeletons of ionic liquids in the preparation of environmentally friendly solvents in organic synthesis, electrolytes, liquid crystals, and so on. Therefore, many reports of the synthesis of imidazole derivatives have appeared. Among those reports, the oxidative conversion of imidazoline into imidazole is shown to be a general and reliable method of synthesis. Encouraged by these reports, as an extension of our activated carbon—molecular oxygen system, we examined the oxidation of imidazolines to imidazoles. Starting 2-arylimidazolines were prepared according to two reported methods. One is the reaction of nitrile with ethylenediamine in the presence of a catalytic amount of S, and the other is the reaction of aldehyde with ethylenediamine in the presence of a stoichiometric amount of NBS or of molecular iodine in the presence of K_2_CO_3_. 2-Arylimidazolines prepared by the above methods were treated with 100 wt% activated carbon (Shirasagi KL, Japan EnviroChemicals, Ltd.) in xylene at 120 ºC under an oxygen atmosphere. The results in Table 5 clearly show that the combination of activated carbon and oxygen accelerated the oxidative conversion of imidazolines into the corresponding imidazoles. As shown in Table 5, a variety of imidazolines having aryl groups at the 2-position were smoothly converted into the corresponding imidazoles in good to high yields. We were also able to confirm that the reuse of activated carbon was possible.

**Table 5:** Oxidative aromatization of 2-arylimidazolines to the corresponding imidazoles.^a^ 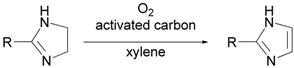

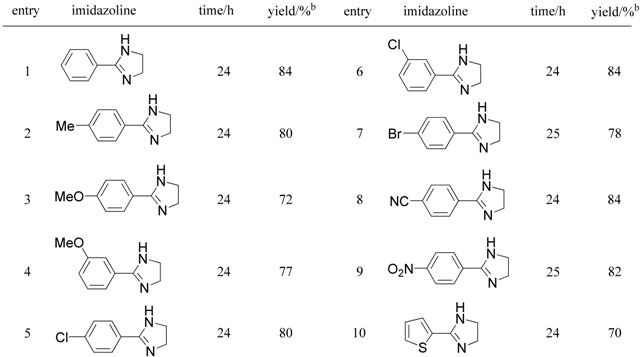

^a^ All reactions were carried out using 100 weight% of activated carbon (Shirasagi KL) in xylene at 120 °C.

### 2.4. Oxidative conversion of functionalized 3,4-dihydropyrimidin-2(1H)-ones into the corresponding Pyrimidin-2(1H)-ones using the activated carbon—molecular oxygen system *[30]*

The functionalized pyrimidin-2(*1H*)-one moiety occurs in a number of natural and pharmaceutical compounds. Because of this, several methods have been developed for the synthesis of these moieties. The oxidative aromatization of dihydroheteroaromatic compounds to aromatic compounds should be an alternative and attractive method, particularly for the synthesis of functionalized pyrimidin-2(*1H*)-ones because 3,4-dihydropyrimidin-2(*1H*)-ones (DHPMs) were easily prepared by the Biginelli reaction [31,32], which involves the condensation of a variety of aldehydes, β-keto esters, and urea. Recently, Yamamoto and coworkers reported the oxidative dehydrogenation of dihydropyrimidinones and dihydropyrimidines with a catalytic amount of Cu salt, K_2_CO_3_, and *tert*-butylhydroperoxide (TBHP) [33]. Here, we report the efficient synthesis of functionalized pyrimidin-2(*1H*)-ones by oxidative aromatization of DHPMs using the activated carbon—molecular oxygen system. 

First, we examined the effect of the amount of activated carbon (Charcoal Activated, TCI) in xylene at 120 ºC for 26 h. As shown in entry 1 of Table 6, when the reaction was carried out in the absence of activated carbon, only 32% of ethyl 4,6-diphenyl-pyrimidin-2(*1H*)-one-5-carboxylate was obtained from the corresponding DHPM. The yield increased to 70% in the presence of 50 wt% activated carbon under the same reaction conditions. When 100 wt% activated carbon was used, the product was obtained in 78% yield. Therefore, other 3,4-dihydropyrimidin-2(*1H*)-ones were employed under an oxygen atmosphere at 120 ºC for 20-48 h using 100 wt% activated carbon. A variety of 3,4-dihydropyrimidin-2(*1H*)-ones were converted into the corresponding pyrimidin-2(*1H*)-ones with the aid of activated carbon under an oxygen atmosphere, as shown in Table 6. The presence of an aryl group at 4- and 6-positions is essential to obtain high yields of the oxidation products.

**Table 6:** Oxidative conversion of functionalized 3,4-dihydropyrimidin-2(*1H*)-ones into the corresponding pyrimidin-2(*1H*)-ones.^a^ 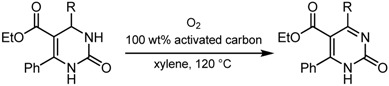

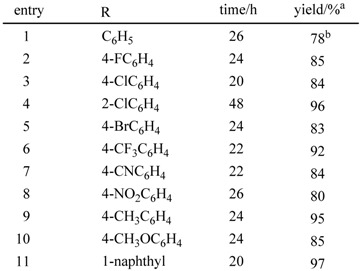

^a^ Isolated yield after recrystallization unless otherwise noted; b Isolated yield after silica-gel column chromatography.

With regard to the structures of the products, we confirmed the structure of ethyl 4,6-diphenyl-pyrimidin-2(*1H*)-one-5-carboxylate by single-crystal X-ray diffractometry, and found that this compound had formed a dimer by assuming the keto form, utilizing the stabilization of the hydrogen bond. Various 3,4-dihydropyrimidin-2(*1H*)-ones were easily prepared by the Biginelli reaction, prooving that the present method is a general and versatile tool for the synthesis of a variety of functionalized pyrimidin-2(*1H*)-ones.

### 2.5. Synthesis of 2-arylbenzoxazoles, 2-arylimidazoles, and 2-arylbenzothiazoles *[34,35]*

We then investigated the synthesis of 2-arylbenzoxazoles, 2-arylimidazoles, and 2-aryl-benzothiazoles. 2-Benzoxazole, -benzimidazole, and -benzothiazole ring moieties are often found in compounds that exhibit antitumor, antimicrobial, and antiviral activities. There are two general methods for the synthesis of 2-substituted benzoxazoles. One method is the coupling of 2-amino-phenols with carboxylic acid derivatives, which is catalyzed by strong acids or requires microwave conditions [36]. The second method is the oxidative cyclization of phenolic Schiff bases derived from the condensation of 2-aminophenols and aldehydes. However, all previously reported oxidants such as DDQ, Mn(OAc)_3_, PhI(OAc)_2_, BaMnO_4_, NiO_2_ and Pb(OAc)_2_ are required in stoichiometric or excess amounts relative to their respective substrates. Recently, the palladium-catalyzed coupling reaction of bromo- or iodobenzene with benzimidazole or benzoxazole was reported [37].

In seeking a system for the synthesis of 2-arylbenzoxazoles by the oxidative cyclization of phenolic Schiff bases, we screened various catalysts and solvents and found that 2-(4’-methoxyphenyl)-benzoxazole was obtained on treatment of 2-(4’-methoxybenzylideneamino)phenol with activated carbon under an oxygen atmosphere by oxidative cyclization in *m*-xylene. As shown in Table 7, the reaction took place in the presence of only activated carbon (Darco^®^ KB) under oxygen at atmospheric pressure (entry 1) or even under air. Thus, palladium is not necessary for this transformation while the presence of activated carbon is crucial.

**Table 7:** Effect of activated carbon. 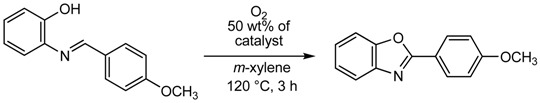

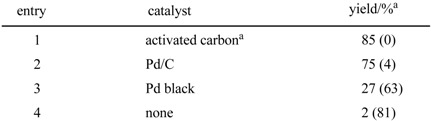

^a^ ^1^H-NMR analysis. The values in parentheses indicate the yield of recovered Shiff base; b Darco® KB (Aldrich Inc.)

Furthermore, we found that it is not necessary to prepare Schiff bases in advance. We can use equimolar amounts of 2-aminophenols bearing substituents and aldehydes as starting materials in the presence of activated carbon. This direct synthesis starting from substituted 2-aminophenols and aldehydes should be synthetically useful and practical. A variety of 2-aminophenol derivatives and aldehydes can be used in this oxidativecyclization via the formation of Schiff bases, which leads to the direct synthesis of 2-arylbenzoxazoles (Table 8).

**Figure 1:**
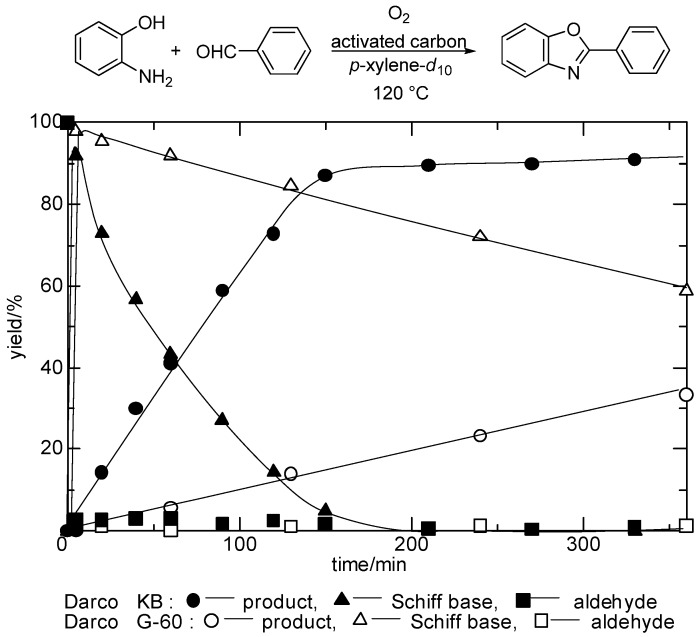
Time course of 2-phenylbenzoxazole, Schiff base, and aldehyde using activated carbon as catalysts which have different properties as shown in Table 9, were employed. Reactions were performed using 2-aminophenol (0.5 mmol), benzaldehyde (0.5 mmol), and activated carbon (50 mg) in *p*-xylene-d_10_ (2 mL) at 120 °C under O_2_. Yields were determined by ^1^H-NMR analyses. Anthracene (0.1 mmol) was used as internal standard.

We next examined the direct synthesis of 2-phenylbenzoxazole from 2-aminophenol and benzaldehyde by using three types of activated carbon with different surface areas (Darco^®^ KB, 1500 m^2^/g; Darco^®^ KB-B, 1500 m^2^/g; and Darco^®^ G-60, 600 m^2^/g), to determine whether there were any differences in reactivity. In each case, we plotted the yields of 2-phenylbenzoxazole (product), Schiff base (intermediate), and aldehyde (starting material) versus time. Figure 1 shows the reaction profile only the case of Darco^®^ KB and Darco^®^ G-60 for simplicity (the results of the case of Darco^®^ KB-B was similar with the case of Darco^®^ KB, so we omitted the line of the result of Darco^®^ KB-B in the figure). Darco^®^ KB and Darco^®^ KB-B exhibited higher activity than Darco^®^ G-60 did. In the reactions using Darco^®^ KB and Darco^®^ KB-B, it took approximately 3 h to obtain the product in 90% yield. However, when Darco^®^ G-60 was used, the product was obtained in less than 20% yield in the same reaction time. The Schiff base was formed very rapidly and the rate-determining step was found to be oxidative cyclization.

**Table 8:** Direct synthesis of 2-arylbenzoxazoles, 2-arylimidazoles, and 2-arylbenzothiazoles. ^a^ 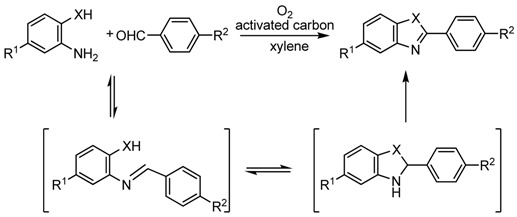

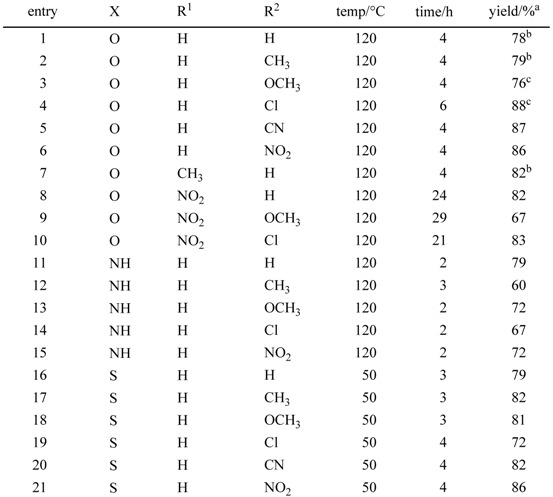

^a^ Isolated yield recrystallization unless otherwise noted; ^b^ Isolated yield by silica-gel column chromatography; ^c^ ^1^H-NMR analysis.

The activated carbon—molecular oxygen system was applied to the synthesis of 2-arylbenzimidazoles (entries 11–5). The coupling of 1,2-phenylenediamine with substituted benzaldehyde proceeded smoothly to give 2-phenylbenzimidazole derivatives in 60%–79% yield. 2-Arylbenzothiazoles and their derivatives have attracted much attention not only in pharmacy but also in material science. Many synthetic procedures for these compounds have been reported, with oxidative cyclization of the corresponding Schiff base being the most generally used. A variety of 4-substituted benzaldehydes reacted with 2-aminobenzenethiol to produce the corresponding 2-arylbenzothiazoles in the presence of activated carbon (Shirasagi KL; Japan EnviroChemicals, Ltd. or Darco^®^ KB; Aldrich, Inc.) under an oxygen atmosphere in high yields (entries 16–21). The reactions occurred at 50 ºC under mild conditions compared to those present in the synthesis of 2-arylbenzoxazoles and 2-arylbenzimidazoles. The reaction also proceeded under an air atmosphere instead of oxygen, although it took longer: examples are the reactions of 2-aminobenzenethiol with benzaldehyde (50 ºC, 13 h, 82%), 4-methylbenzaldehyde (50 ºC, 13 h, 82%), and 4-chlorobenzald-ehyde (50 ºC, 13 h, 76%). 

### 2.6. Role of activated carbon: How does activated carbon promote the oxidation reaction? *[38]* —investigation of the effect of metal contaminants

We investigated the role of activated carbon in the reaction of 2-aminophenol with 4-methoxy-benzaldehyde via oxidative cyclization of the intermediate 2-[(4-methoxybenzylidene)amino]-phenol. First, we examined the effect of metal contaminants in the activated carbon on reactivity in the oxidative aromatization, employing six tailor-made activated carbons (samples A to F). These activated carbons were made from wood (sample A), coal (sample B), and coconut shell (samples C, D, E, and F). As for the activation method, we used two Darco^®^ KB and G-60, methods: one was chemical activation using ZnCl_2_ (samples A, E, and F) and the other was steam activation (samples B, C, and D).

Samples A—F were treated with 3% HCl aq. for 10 min and then with boiling water ten times. The reaction yields using unwashed activated carbons (samples A to F) were compared with those using washed activated carbons (samples A’ to F’), respectively. Metal contaminants in activated carbon, such as Zn, Co, Mn, Fe, Cr, Mg, V, Cu, Pd, Ca, Na, and K, were measured by ICP-AES in washed and unwashed samples. 

**Table 9:** Properties of activated carbons.

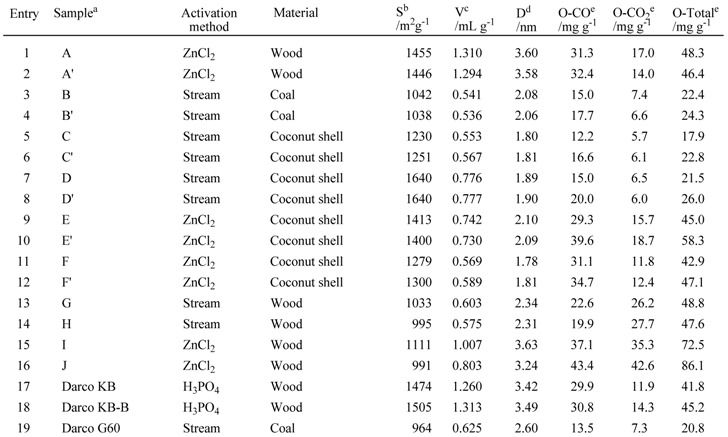

^a^ X´ indicates washed sample of X with H2O; ^b^ Specific surfaces area; ^c^ Pore volume; ^d^ Mean pore volume; ^e^ Amount of oxygen.

**Figure 2:**
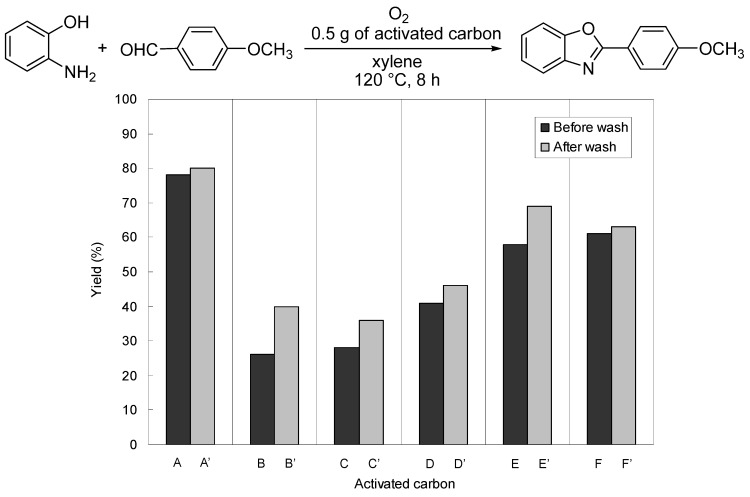
Reaction yields using washed and unwashed activated carbons.

We confirmed that the amounts of those metal contaminants decreased after washing. The yields of 2-(4-methoxyphenyl)benzoxazole using washed activated carbons were higher than those using unwashed activated carbons (Figure 2). Therefore, we concluded that the driving factor of this oxidation system was not the metal contaminants in activated carbon but other factors. The reason why washed activated carbons exhibited higher yields than unwashed activated carbons will be discussed in the next section.

### 2.7. Role of activated carbon [38]—investigation of the effects of specific surface area, pore volume, mean pore diameter, and surface functionality

Then, we investigated the role of activated carbons in the reaction of 2-aminophenol with 4-methoxybenzaldehyde via oxidative cyclization of intermediate 2-[(4-methoxybenzylidene)amino]-phenol. To examine the effects of specific surface area, pore volume, mean pore diameter, and surface functionality, we employed nineteen activated carbons, including sixteen tailor-made activated carbons and three commercially available activated carbons. These activated carbons were obtained using different raw materials and activation methods, and therefore, they have different specific surface areas, pore volumes, mean pore diameters, and surface oxygen group contents and amounts, as summarized in Table 9. Specific surface area was measured using the Brunauer-Emmett-Teller (BET) method [39] with nitrogen gas as adsorbate. Pore volume and mean pore diameter were calculated according to the Cranston-Inkley (CI) method [40] from the volume of adsorbed N_2_. Oxygen content was determined by GC measurement of evolved gases, such as CO and CO_2_, at 900 ºC, which originated in decomposed surface oxygen groups of 1 g of activated carbon.

**Figure 3:**
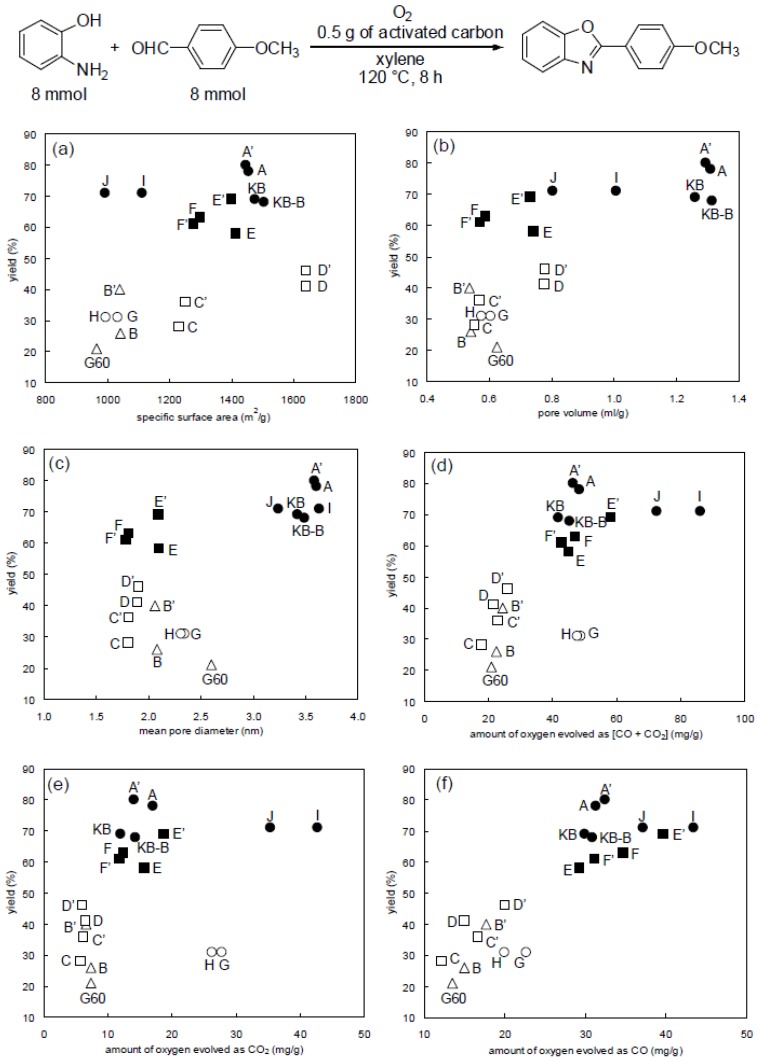
Effect of properties of activated carbons listed in Table 2 on reaction yields. (a) Specific surface area, (b) pore volume, (c) mean pore diameter, (d) amount of oxygen evolved as [CO + CO_2_], (e) amount of oxygen evolved as CO, and (f) amount of oxygen evolved as CO_2_. ● Chemical activation (wood), ■ chemical activation (coconut shell), ○ steam activation (wood), □steam activation (coconut shell), ∆ steam activation (coal).

Figure 3 shows the relationship between the properties of activated carbons and the yields in oxidative aromatization. It is clear that chemically activated carbons (filled symbols ● ■ in Figure 3) had higher reactivity than steam activated carbons (unfilled symbols ○ □ ∆). Then, we turned our attention to surface oxygen groups because the chemical activation method was usually conducted at a lower temperature than the steam activation method and thus more surface oxygen groups remained on the surface of activated carbons obtained by the former method. As shown in Figure 3(f), surface oxygen groups that evolved as CO have the best tendency to increase reaction yields among all the properties examined. The total oxygen amounts evolved as CO and CO_2_ and the oxygen amounts evolved as CO_2_ did not have a strong relationship with the reaction yields [Figure 3 (d), (e)]. Here, CO would be derived from phenol, carbonyl and quinone groups, and CO_2_ would be derived from carboxyl and lactone groups that existed on original activated carbon surfaces. Specific surface area, pore volume, and mean pore diameter appeared to be little related to the reaction yield [Figure 3 (a), (b), (c)].

### 2.8. Aromatization of 9,10-dihydroanthracenes using molecular oxygen promoted by activated carbon [41]

Several methods for the aromatization of polycyclic hydroaromatic compounds, such as substituted 9,10-dihydroanthracenes, have been developed. However, most of them require a stoichiometric or an excess amount of oxidant. Furthermore, in some cases, an extremely high temperature is necessary. Therefore, a more efficient process that uses oxygen as an oxidizing agent and an effective catalyst is desirable from the environmental point of view. Yamada and coworkers have reported oxidative aromatization with oxygen catalyzed by a ruthenium porphyrin complex [42,43]. In this review, we present an environmentally friendly method for the oxidative aromatization of several 9,10-dihydroanthracenes using molecular oxygen and promoted by inexpensive and readily available activated carbon. Mizuno also reported the oxidation of 9,10-dihydroanthracene using the Ru(OH)_x_/Al_2_O_3_ (Ru; 2 mol%) catalyst system at 100 ºC in trifluorotoluene under an oxygen atmosphere [44].

First, we examined the aromatization of 9,10-dihydroanthracene using Pd/C catalyst and found that a reaction using 50 wt% of 10% Pd/C under an oxygen atmosphere in xylene at 120 ºC exhibited high performance in this conversion (93% yield, entry 1 in Table 10). When the reaction was carried out in the presence of 50 wt% Pd black (entry 2), the aromatization proceeded slowly (36% yield) and the starting material was recovered in 63% yield. Based on these results, we assumed that activated carbon might play an important role in the reaction. Actually, the reaction proceeded by employing activated carbon without any palladium source (entry 3), that is, on the treatment of 9,10-dihydroanthracene with 50 wt% activated carbon (Darco^®^ KB, Aldrich, Inc.).

**Table 10:** Aromatization of substituted 9,10-dihydroanthracenes.

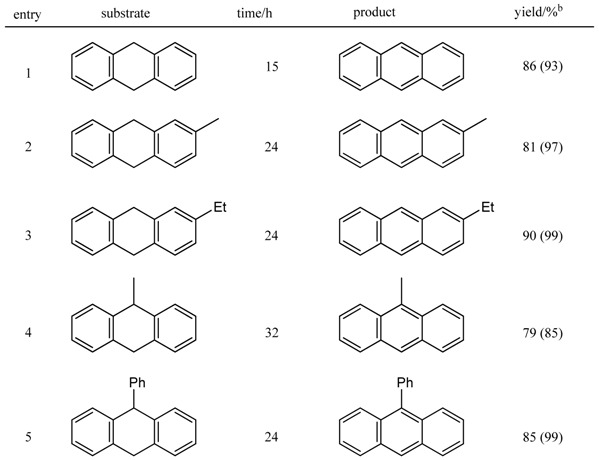

^a^ All reactions were carried out using 200-300 mg of substrate, 50 wt% of activated carbon (Darco®KB, Aldrich Inc.) in xylene (3.5 mL) at 120 °C under oxygen atmosphere; ^b^ Isolated yield by recrystallization; The values in perenttheses indicated the yields determined by ^1^H-NMR analysis.

We then examined the aromatization of 9,10-dihydroanthracene by using three types of activated carbon: Darco^®^ KB, Darco^®^ KB-B, and Darco^®^ G-60 (Table 10). We found that the use of Darco^®^ KB and Darco^®^ KB-B, which had a greater surface area than did Darco^®^ G-60, exhibited higher activity in the conversion of 9,10-dihydroanthracene into the corresponding aromatized anthracene (entries 1 and 2). We measured the metal content in the above three types of activated carbon by ICP-AES. Among the metals detected, we focused on Fe and found that it was present in the following amounts in one gram of activated carbon: Darco^®^ KB, 299 ppm; Darco^®^ KB-B, 98.3 ppm; and Darco^®^ G-60, 175 ppm. Among the activated carbon samples examined, Darco^®^ KB-B contained the least amount of Fe. However, its reactivity was almost the same as that of Darco^®^ KB, which contained the greatest amount of Fe. In these activated carbon samples, the important difference is not the amount of Fe but the surface area: Darco^®^ KB, 1500 m^2^/g; Darco^®^ KB-B, 1500 m^2^/g; and Darco^®^ G-60, 600 m^2^/g (data from Aldrich, Inc.). Therefore, it is assumed that surface area would have a greater effect on reactivity than would the amount of Fe in activated carbon. 

Several examples of the aromatization of 9,10-dihydroanthracene derivatives to the corresponding anthracenes are summarized in Table 10. 9,10-Dihydroanthracenes bearing a variety of substituents were treated with 50 wt% activated carbon (Darco^®^ KB) in xylene under an oxygen atmosphere (120 ºC, 15–2 h) to give the corresponding substituted anthracenes in high yields (79–90%). In conclusion, we have disclosed an extremely simple method of oxidative aromatization to convert 9,10-dihydroanthracenes into the corresponding anthracenes using molecular oxygen promoted by activated carbon.

As described above, the activated carbon—molecular oxygen system was found to be efficient for oxidative aromatization, as summarized below. Compared with previous methods, this method is advantageous in terms of operational simplicity, cost performance, and environmental load.

## 3. Conclusions

A variety of heteroaromatic and aromatic compounds, including substituted pyridines, pyrazoles, benzoxazoles, benzimidazoles, benzothiazoles, 2-substituted imidazoles, indoles, pyrimidin-2(*1H*)-ones, and anthracenes, have been prepared by oxidative aromatization using the activated carbon—molecular oxygen system. 


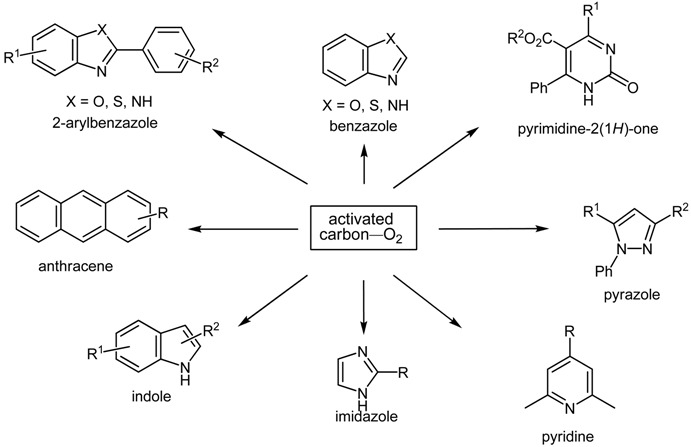


To clarify the reaction mechanism in order to obtain information of the role of activated carbon, we examined oxidative aromatization using more than ten kinds of activated carbon having different surface areas, micropore volumes, and oxygen-containing functional groups. We revealed that the multiplier effect of surface area and the content of the oxygen functional group evolving as CO in micropores and mesopores played a very important role in promoting an efficient reaction. We also found that Shirasagi KL and Darco^®^ KB are more effective than are other activated carbons. Concerning the contamination by trace amounts of metals existing in the starting materials, we concluded that the metal contaminants exert a minimal effect on the oxidation. Functionalized 2-arylbenzoxazoles, 2-arylbenzimidazoles, and 2-arylbenzothiazoles were synthesized via oxidative aromatization of their dihydro compounds using the activated carbon—molecular oxygen system. Detailed mechanistic studies revealed that surface oxygen groups evolving as CO, such as carbonyl groups on the surface of activated carbon, promoted the dehydrogenation step in this activated carbon—molecular oxygen system. Additionally, in the synthesis of 2-arylbenzoxazoles, activated carbon would also promote the cyclization of intermediate Schiff base. It should be mentioned that recovery and reuse of activated carbon were possible.
